# A single amino acid substitution of the human immunodeficiency virus type 1 capsid protein affects viral sensitivity to TRIM5α

**DOI:** 10.1186/1742-4690-7-58

**Published:** 2010-07-07

**Authors:** Ayumu Kuroishi, Katarzyna Bozek, Tatsuo Shioda, Emi E Nakayama

**Affiliations:** 1 Department of Viral Infections, Research Institute for Microbial Diseases, Osaka University, Osaka 565-0871, Japan; 2 Max Planck Institute for Informatics, Campus E1.4, 66123 Saarbrücken, Germany

## Abstract

**Background:**

Human immunodeficiency virus type 1 (HIV-1) productively infects only humans and chimpanzees but not Old World monkeys, such as rhesus and cynomolgus (CM) monkeys. To establish a monkey model of HIV-1/AIDS, several HIV-1 derivatives have been constructed. We previously reported that efficient replication of HIV-1 in CM cells was achieved after we replaced the loop between α-helices 6 and 7 (L6/7) of the capsid protein (CA) with that of SIVmac239 in addition to the loop between α-helices 4 and 5 (L4/5) and *vif*. This virus (NL-4/5S6/7SvifS) was supposed to escape from host restriction factors cyclophilin A, CM TRIM5α, and APOBEC3G. However, the replicative capability of NL-4/5S6/7SvifS in human cells was severely impaired.

**Results:**

By long-term cultivation of human CEMss cells infected with NL-4/5S6/7SvifS, we succeeded in rescuing the impaired replicative capability of the virus in human cells. Sequence analysis of the CA region of the adapted virus revealed a G-to-E substitution at the 116th position of the CA (G116E). Introduction of this substitution into the molecular DNA clone of NL-4/5S6/7SvifS indeed improved the virus' replicative capability in human cells. Although the G116E substitution occurred during long-term cultivation of human cells infected with NL-4/5S6/7SvifS, the viruses with G116E unexpectedly became resistant to CM, but not human TRIM5α-mediated restriction. The 3-D model showed that position 116 is located in the 6^th ^helix near L4/5 and L6/7 and is apparently exposed to the protein surface. The amino acid substitution at the 116^th ^position caused a change in the structure of the protein surface because of the replacement of G (which has no side chain) with E (which has a long negatively charged side chain).

**Conclusions:**

We succeeded in rescuing the impaired replicative capability of NL-4/5S6/7SvifS and report a mutation that improved the replicative capability of the virus. Unexpectedly, HIV-1 with this mutation became resistant to CM TRIM5α-mediated restriction.

## Background

Human immunodeficiency virus type 1 (HIV-1) productively infects only humans and chimpanzees, but not Old World monkeys (OWM) such as cynomolgus (CM) and rhesus (Rh) monkeys [1]. Unlike the replication of simian immunodeficiency virus isolated from macaques (SIVmac), HIV-1 replication is blocked early after viral entry, before the establishment of a provirus in OWM cells [1-3]. To establish a monkey model of HIV-1/AIDS, several viruses that are chimeras of HIV-1 and SIVmac (SHIV) have been constructed and tested for replicative capability in simian cells [4,5]. The host range of HIV-1 was limited because of some intrinsic restriction factors in simian cells, such as ApoB mRNA editing catalytic subunit (APOBEC) 3G [6], cyclophilin A (CypA) [7-9], BST-2 (CD317; tetherin) [10,11] and TRIM5α, a member of the tripartite motif (TRIM) family proteins [12]. Rh and CM TRIM5α restrict HIV-1, but not SIVmac [13,14]. A lack of functional TRIM5α expression in pig-tailed monkey enabled Hatziioannou et al. to construct a SHIV strain that differs from HIV-1 only in the *vif *gene and can efficiently replicate in pig-tailed monkeys [15]. Although this virus was designed to escape from monkey APOBEC3G mediated restriction, this virus failed to grow in Rh and CM cells. Kamada et al. attempted to evade the restrictions mediated by CypA in OWM cells by replacing the loop between α-helices 4 and 5 (L4/5) of the HIV-1 capsid (CA) with that of SIVmac in addition to *vif *because CypA fails to bind to the L4/5 of SIVmac. However, this was not enough to escape from TRIM5α-mediated restriction [16].

TRIM5α consists of RING, B-box 2, coiled-coil, and SPRY (B30.2) domains [17]. TRIM5α recognizes the multimerized CA of an incoming virus by its α-isoform specific SPRY domain [18-20]. Studies on chimeric TRIM5αs have shown that the determinant of the species-specific restriction against viral infection resides in the variable regions of the SPRY domain [21,22]. On the other hand, we previously identified a single amino acid of the surface-exposed loop between α-helices 6 and 7 (L6/7) of the HIV-2 CA as a determinant of the susceptibility of HIV-2 to CM TRIM5α[23]. On the basis of this finding, we have succeeded in improving simian-tropic HIV-1, which was generated by Kamada et al. [5], by replacing L6/7 of CA with those of SIVmac239 in addition to L4/5 and vif [24]; the new resultant virus has more efficient replication in CM cells. The resultant virus, NL-ScaVR6/7S, showed efficient replicative capability in CM cells; however, the replicative capability of this virus in human cells was severely impaired.

In the present report, we describe our efforts to rescue the impaired replicative capability of NL-ScaVR6/7S after long-term cultivation in human CEMss cells, and we report on the amino acid mutation that improved the replicative capability of this virus.

## Materials and methods

### Viral adaptation

For viral adaptation in human cells, 100 ng of p24 of NL-ScaVR6/7S [24], renamed in this report as NL-4/5S6/7SvifS, was inoculated into 1 × 10^6 ^of human T cell line CEMss cells. The infected culture was gradually expanded to keep the cell concentration at 1 × 10^6^/mL. The culture supernatants were collected periodically, and p24 levels were measured with an ELISA kit (ZeptoMetrix, Buffalo, NY). Virus in the culture supernatant at day 42 after infection was designated NL-4/5S6/7SvifSd42, and inoculated into fresh CEMss cells. Six days after re-infection, the matrix (MA)-CA region of the integrated provirus was amplified by PCR from the genomic DNA of infected cells and cloned into pCR 2.1-TOPO vector (Invitrogen, Carlsbad, CA) to generate pTopo-MA-CAadp42. Nucleotide sequences of 6 independent clones were determined by ABI Prism 3100 Genetic Analyzer (Applied Biosystems, USA).

### DNA constructions

**Figure 1:**
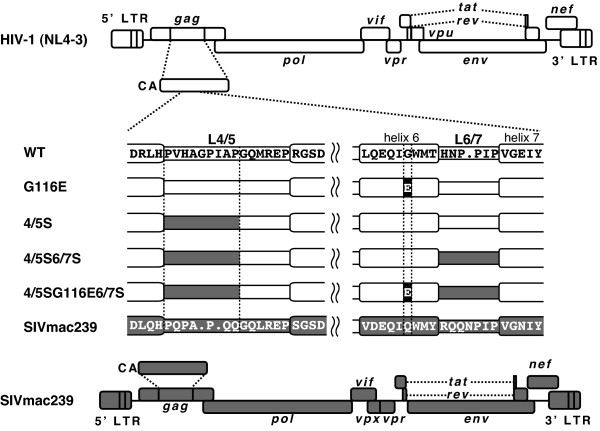
**Schematic representation of HIV-1 derivatives**. White and gray bars denote HIV-1 (NL4-3) and SIVmac239 sequences, respectively. "E" indicates the amino acid residue at the 116th position of the capsid protein (CA).

The HIV-1 derivatives were constructed on a backbone of infectious molecular clone NL4-3 [25]. To introduce a glycine (G)- to-glutamic acid (E) substitution at the 116^th ^position of CA (G116E) into NL-4/5S6/7SvifS, the 0.5 kb SpeI-ApaI fragment, which corresponds to the N-terminus of the CA including the 116th position and L6/7, of pTopo-MA-CAd42 was transferred into NL-4/5S6/7SvifS to generate NL-4/5SG116E6/7SvifS. The G116E substitution was also introduced into NL4-3 and NL-SVR (renamed NL-vifS in this report) by site-directed mutagenesis with the PCR-mediated overlap primer extension method. Resultant constructs were designated NL-G116E and NL-G116EvifS, respectively (Figure 1). To construct the wild type and mutant HIV-1 clones expressing green fluorescence protein (GFP), the 1.3 kb BssHII-ApaI fragment of NL-G116E, NL-4/5S6/7SvifS, or NL-4/5SG116E6/7SvifS, which corresponds to the MA and CA, was transferred to NL-Nhe GFP, in which the *env *gene was interrupted; and the GFP gene was inserted into the *nef *region. Resultant constructs were designated G116E-GFP, 4/5S6/7S-GFP, and 4/5SG116E6/7S-GFP, respectively. To construct the lentivector expressing GFP under the control of cytomegalovirus promoter, we replaced the Eco RI-Apa I fragment corresponding to MA and CA of the pMDLg/p.RRE packaging vector [24,26,27] with that of NL-G116E, and designated the resultant construct as pMDLg/p.RRE-G116E.

#### Cells and virus propagation

The human kidney adherent 293T cells were cultured in Dulbecco's modified Eagle medium supplemented with 10% heat-inactivated fetal bovine serum (FBS). The human T cell lines CEMss and MT4 were maintained in RPMI 1640 medium supplemented with 10% FBS. Virus stocks were prepared by transfection of 293T cells with HIV-1 NL4-3 and its derivatives using the calcium phosphate co-precipitation method. Viral titers were measured with an ELISA kit.

Sendai viruses (SeV) expressing CM TRIM5α, human TRIM5α, Rh TRIM5α, and CM TRIM5α without the SPRY domain [CM-SPRY (-)] were described previously [18,23,28].

A cell line stably expressing CM or humanTRIM5α was established as described previously [18]. Briefly, a pCEP4 plasmid (Invitrogen) encoding CM or human TRIM5α fused with HA tag in its C-terminus was transfected into TK-ts13 hamster cells. Transfected cells were then cultured in the presence of 0.3 mg/ml of hygromycin B (Gibco) for 14 days to remove untransfected cells. The expression of TRIM5α was confirmed by Western blot analysis of cell lysate with anti-HA antibody (HA High Affinity, Roch).

#### Viral infections

CEMss or MT4 cells (1 × 10^5^) were infected with 20 ng of p24 of NL-4/5SvifS, NL-4/5S6/7SvifS, or NL-4/5SG116E6/7SvifS. The culture supernatants were collected periodically, and p24 levels were measured with an ELISA kit. To analyze the viral sensitivity to TRIM5α, 1 × 10^5 ^CEMss cells were first infected with SeV expressing each of the TRIM5αs at a multiplicity of infection of 10 plaque-forming units per cell and incubated at 37°C for 9 hours. Cells were then superinfected with 20 ng of p24 of HIV-1 NL4-3 or its derivatives. The culture supernatants were collected periodically, and the levels of p24 were measured with an ELISA kit.

For the single-round infection assay, CEMss or canine Cf2Th cells were infected with SeV expressing TRIM5α as described above, and super-infected with vesicular stomatitis virus glycoprotein (VSV-G) pseudotyped HIV-1 clones expressing GFP. In case of TK-ts13 hamster cells stably expressing CM, human or CM-SPRY(-) TRIM5α, cells were infected with VSV-G pseudotyped lentivector expressing GFP under the control of cytomegalovirus promoter. Two days after infection, the cells were fixed by formaldehyde, and GFP expressing cells were counted with a flow-cytometer. The percentage of the GFP-positive cells in the presence of TRIM5α was divided by the percentage of GFP-positive cells in the presence of CM-SPRY (-) to define the percent of infection. The differences in percent infection between WT-GFP and G116E-GFP, or 4/5S6/7S-GFP and 4/5SG116E6/7S-GFP were statistically evaluated by using the unpaired t test.

#### Particle purification and Western blotting

The culture supernatants of 293T cells transfected with plasmids encoding HIV-1 NL4-3 derivatives were clarified by low-speed centrifugation. Nine milliliters of the resultant supernatants were layered onto a 2 mL cushion of 20% sucrose in phosphate buffered saline (PBS) and centrifuged at 35,000 rpm for 2 hours in a Beckman SW41 rotor. After centrifugation, the virion pellets were resuspended in PBS, and p24 antigen concentrations were measured by ELISA. Fifty nanograms of p24 of HIV-1 derivatives were applied to SDS-polyacrylamide gel electrophoresis, and the virion-associated proteins were transferred to a PVDF membrane. CA and CypA proteins were visualized with the anti-p24 antibody (Abcam) and anti-CypA antibody (Affinity BioReagents, Golden, CO), respectively.

#### Modeling

The structure of the N-terminal domain of the HIV-1 CA protein (PDB number 1GWP) [29] was used as a template for building the domain model with the G116E substitution. The model was built using Modeller 9v4 [30] and visualized with PyMOL v1.0r2 (The PyMOL Molecular Graphics System, http://pymol.sourceforge.net/).

## Results

### A virus with SIVmac CA L4/5, L6/7, and vif gained efficient replicative capability after adaptation in human T cell line

**Figure 2:**
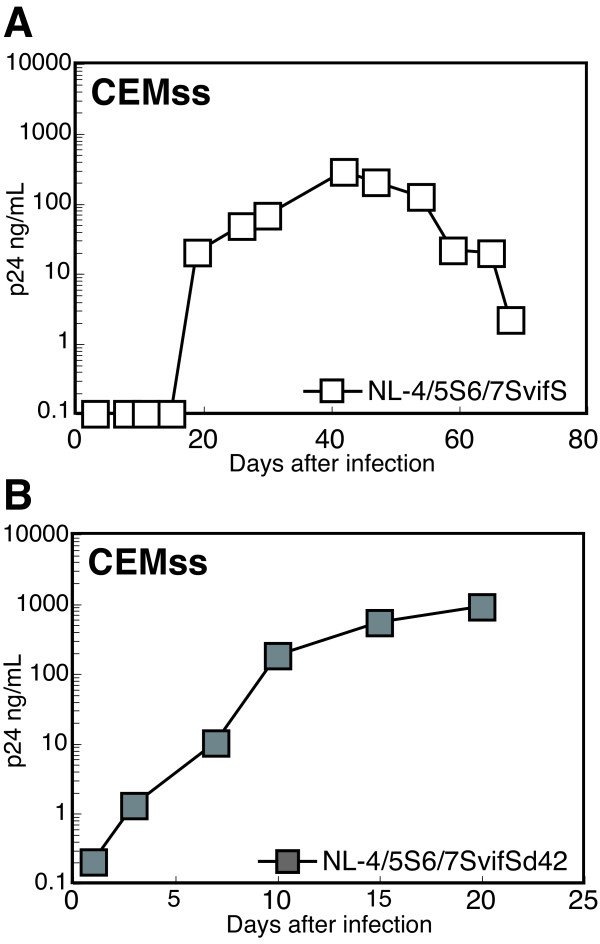
**Adaptation of HIV-1 derivatives to human cells**. (A) NL-4/5S6/7SvifS, a virus with the SIVmac L4/5, L6/7, and *vif *was inoculated into CEMss cells, and culture supernatants were periodically assayed for the levels of p24. (B) Virus in the culture supernatant on day 42 after infection (NL-4/5S6/7SvifSd42) was inoculated into fresh CEMss cells.

We previously reported that in addition to L4/5 of the CA and *vif*, L6/7 of the SIVmac CA is important for the efficient replication of HIV-1 derivatives in CM cells [24]. While introduction of SIVmac L6/7 into an HIV-1 derivative improved viral growth in CM cells, the replicative capability in human cells was greatly attenuated. To gain more insight into the effects of the L6/7 replacement on viral replication, we attempted to rescue the impaired replicative capability by long-term cultivation in human CEMss cells. NL-ScaVR6/7S, a virus with SIVmac L4/5, L6/7, and *vif *renamed NL-4/5S6/7SvifS in the present study, was inoculated into CEMss cells; and culture supernatants were periodically assayed for the levels of p24. Progeny virions were first detectable on day 20 after infection and reached a peak titer on day 42 (Figure 2A). The virus in the culture supernatant on day 42 was designated NL-4/5S6/7SvifSd42 and inoculated into fresh CEMss cells (Figure 2B). This time, the progeny virus was detectable on day 3 and reached a peak on day 20, suggesting that the NL-4/5S6/7SvifSd42 gained certain mutation(s) that overcame the attenuated replicative capability. Therefore, we amplified by PCR and cloned the integrated proviral DNA corresponding to the MA and CA regions in the NL-4/5S6/7SvifSd42-infected CEMss cells on day 6. Nucleotide sequence analysis of the resultant clones revealed that 6 out of 6 independent clones carried a single nucleotide substitution at the 347th position of the CA region, resulting in a G-to-E substitution at the 116^th ^position of the CA (G116E).

Analysis of 95 HIV-1 strains in the Los Alamos HIV sequence databases http://www.hiv.lanl.gov/, including subtypes A to K of group M, revealed that there was no HIV-1 strain carrying glutamic acid at the 116th position of the CA, although this position was occupied with variable amino acid residues (35 strains carried glycine; 36, alanine; 9, threonine; 7, arginine; 6, glutamine; 1 each, isoleucine or aspartic acid).

### A single amino acid substitution in CA rescued impaired replicative capability in human cells

**Figure 3:**
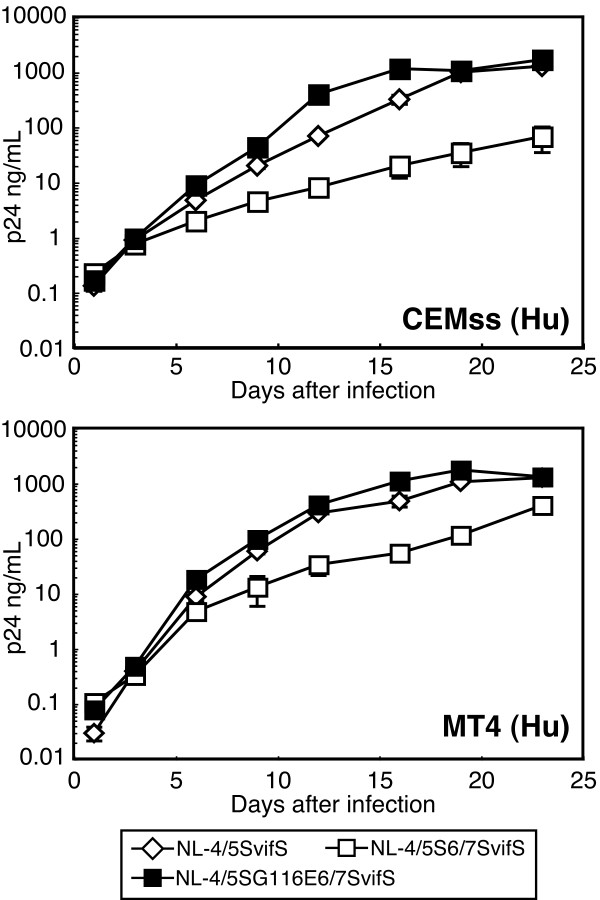
**Replication properties of HIV-1 derivatives**. Equal amounts of NL-4/5SvifS (white diamonds: virus with SIVmac L4/5 and *vif*), NL-4/5S6/7SvifS (white squares: virus with SIVmac L4/5, L6/7, and *vif*), or NL-4/5SG116E6/7SvifS (black squares: virus with the additional replacement of the 116th amino acid Gly with Glu in NL-4/5S6/7SvifS) were inoculated into human CEMss or MT4 cells, and culture supernatants were collected periodically. The levels of p24 antigen were measured by ELISA. A representative of three independent experiments is shown.

To determine whether the single amino acid substitution at the 116th position of the CA improved the replicative capability of NL-4/5S6/7SvifS in human cells, we introduced the G116E mutation into NL-4/5S6/7SvifS. Resultant viruses were designated NL-4/5SG116E6/7SvifS and inoculated into human CEMss or MT4 cells together with their parental viruses to analyze their replicative capability (Figure 3). As described previously [24], NL-4/5S6/7SvifS showed less efficient growth in both CEMss and MT4 human cell lines than did NL-4/5SvifS. NL-4/5SG116E6/7SvifS could grow more efficiently in both human cells than did the parental NL-4/5S6/7SvifS, and its growth was comparable to that of NL-4/5SvifS (Figure 3). These data suggest that the rescued replicative capability of NL-4/5S6/7SvifSd42 in human cells (Figure 2) was the result, at least partly, of the acquisition of the G116E substitution in the CA.

### The amino acid residue at the 116th position of the CA affects viral growth in the presence of TRIM5α

**Figure 4:**
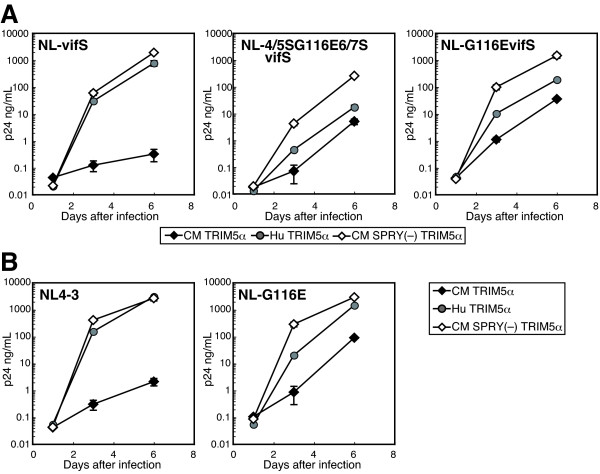
**Viral growth in the presence of TRIM5α**. CEMss cells were infected with recombinant Sendai virus (SeV) expressing CM (black diamonds), human (gray circles), or CM-SPRY (-) (white diamonds) TRIM5α. Nine hours after infection, cells were superinfected with the indicated HIV-1 derivatives. Culture supernatants were separately assayed for levels of p24. Error bars show actual fluctuations between levels of p24 in duplicate samples. A representative of three independent experiments is shown.

We previously reported that NL-4/5S6/7SvifS could grow in CM cells [24], but failed to directly demonstrate that this virus could grow in human cells expressing CM TRIM5α because of its impaired growth capability in human cells. Because the G-to-E substitution at the116th amino acid position rescued the impaired growth capability of NL-4/5S6/7SvifS in human cells, we investigated whether NL-4/5SG116E6/7SvifS could grow in human cells expressing CM TRIM5α (Figure 4A). For TRIM5α expression, we used SeV expressing CM TRIM5α or human TRIM5α. SeV expressing CM-SPRY (-) was used as a TRIM5α-negative control [31]. NL-SVR, a virus with SIVmac *vif *renamed NL-vifS in the present study, did not grow at all in CEMss cells expressing CM TRIM5α. In contrast, NL-4/5SG116E6/7SvifS could grow in CEMss cells expressing CM TRIM5α (Figure 4A), although the viral titers were less than 10% of those in the absence of TRIM5α. Similarly, the human cell-adapted virus NL-4/5S6/7SvifSd42 could also grow in CEMss cells expressing CM TRIM5α (data not shown). To clarify the impact of the single G-to-E substitution in CA on virus growth in the presence of CM TRIM5α, we next introduced a G116E substitution in NL-vifS to generate NL-G116EvifS. We first anticipated that this virus would fail to replicate in CEMss cells expressing CM TRIM5α. Contrary to our expectations, however, this virus grew in the presence of CM TRIM5α to levels similar to those of NL-4/5SG116E6/7SvifS. This result indicates that the single amino acid residue in CA could affect the viral sensitivity to CM TRIM5α mediated restriction. To exclude any possible effect of SIVmac vif in NL-G116EvifS on TRIM5α-mediated restriction, we constructed NL-G116E, a virus with a single amino acid substitution at the 116th position of the CA only (Figure 4B). This virus could also replicate in CEMss cells expressing CM TRIM5α, confirming the importance of the 116th amino acid residue of the CA in TRIM5α-mediated restriction.

With respect to viral sensitivity to human TRIM5α, the growth of both NL-G116EvifS and NL-4/5SG116E6/7SvifS was slightly impaired compared with that of NL-vifS in CEMss cells over-expressing human TRIM5α. The growth of the NL4-3 virus was not affected by human TRIM5α, while that of NL-G116E was slightly suppressed by human TRIM5α. These results suggest that the viruses with G116E substitution were more sensitive to human TRIM5α although the G116E substitution occurred during long-term cultivation of human cells infected with NL-4/5S6/7SvifS. This excludes a possibility that the improved replicative capability of human cell-adapted virus is the result of escape from human TRIM5α-mediated restriction.

### A G116E substitution affects viral sensitivity to CM TRIM5α-mediated restriction in a single-round infection assay

**Figure 5:**
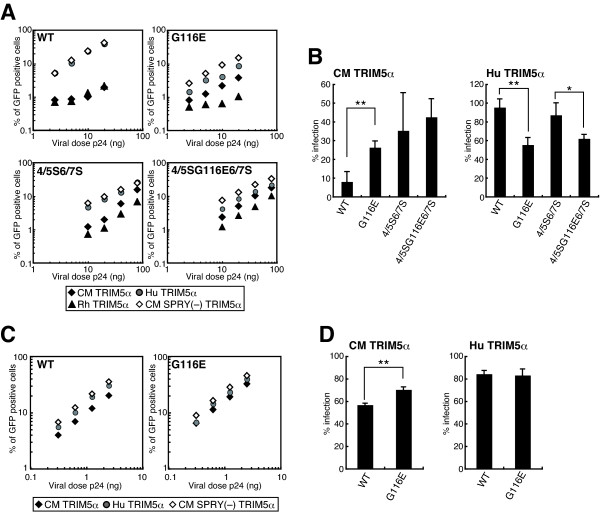
**Viral sensitivity to TRIM5α-mediated restriction in a single-round infection assay**. CEMss cells were infected with SeVs expressing CM (black diamonds), human (Hu: gray circles), rhesus monkey (Rh: black triangles), or CM-SRPY(-) (white circles) TRIM5α. The cells were then superinfected with serially diluted HIV-1-GFP with the indicated CA. (B) The percentage of the GFP-positive cells in the presence of TRIM5α was divided by the percentage of GFP-positive cells in the presence of CM SPRY (-) TRIM5α to define percent infection. The differences in percent infection between WT-GFP and G116E-GFP, or 4/5S6/7S-GFP and 4/5SG116E6/7S-GFP were statistically evaluated by unpaired *t *test (*: *P *< 0.05, **; *P *< 0.01). The representative results of three independent experiments with similar results are shown. (C) TK-ts13 cells stably expressing CM (black diamonds), human (Hu: gray circles), or CM-SRPY(-) (white circles) TRIM5α were infected with serially diluted lentivector expressing GFP under the control of cytomegalovirus promoter with the indicated CA. (D) The percentage of the GFP-positive cells in the presence of TRIM5α was divided by the percentage of GFP-positive cells in the presence of CM SPRY (-) TRIM5α to define percent infection. The differences in percent infection between the wild type and G116E were statistically evaluated by unpaired *t *test (**; *P *< 0.01). The representative results of three independent experiments with similar results are shown.

The assay described in Figures 3 and 4 investigated the effects of CM TRIM5α on the multi-step growth of the viruses. To evaluate the effects of CM TRIM5α on the early steps of viral infection, we performed a single-round infection assay. The fragment of NL-G116E, NL-4/5S6/7SvifS, or NL-4/5SG116E6/7SvifS corresponding to the MA and CA was transferred to an env-deleted HIV-1 genomic clone, which express GFP after infection. VSV-G pseudotyped wild type and mutant HIV-1 GFP viruses were inoculated into CEMss cells expressing TRIM5α and GFP positive cells were counted 2 days after infection (Figure 5A). Because the replicative capability of NL-4/5S6/7SvifS in human cells was lower than that of the wild type virus as described above, it was highly likely that the infectivity of 4/5S6/7S-GFP would also be lower than those of WT-GFP and G116E-GFP. Therefore, we used higher input doses of 4/5S6/7S-GFP and 4/5SG116E6/7S-GFP than those of WT-GFP and G116E-GFP. Ratios of the GFP-positive percentage of cells expressing CM TRIM5α to those of cells expressing non-functional CM-SPRY(-)-TRIM5α are shown as percent of infection in Figure 5B. The percent of infection was relatively constant among the different input doses. Consistent with the results that NL-G116E could replicate in human cells expressing CM TRIM5α (Figure 4B), the GFP-expressing virus with the G116E substitution was more resistant to CM TRIM5α-mediated restriction than the wild type virus, while both viruses were completely restricted by Rh TRIM5α (Figure 5A, Figure 5B left). Similar results were obtained when we used Cf2Th canine cells lacking endogenous TRIM5α expression, although the number of GFP-positive cells was less than that of CEMss cells (data not shown). These results in the single-round infection assay clearly confirmed our results in the live virus replication experiments showing that the G116E substitution conferred resistance against CM-TRIM5α-mediated restriction. While both the GFP-expressing viruses with the 4/5S6/7S (4/5S6/7S-GFP and 4/5SG116E6/7S-GFP) were resistant to CM TRIM5α, an additional effect of the G116E substitution was not observed (Figure 5B, left). To examine the effect of G116E substitution in cells with more physiological levels of TRIM5α expression, we established TK-ts13 hamster cells stably expressing CM or human TRIM5α and inoculated lentivector expressing GFP under the cytomegalovirus promoter into these cells. As shown in Figure 5C and 5D, the GFP expression from the lentivector with the wild type CA was suppressed in TK-ts13 cells expressing CM TRIM5α, although the levels of suppression were less than those in Figure 5B due to lower levels of CM TRIM5α expression. As expected, the lentivector with the G116E substitution showed reduced suppression by CM TRIM5α compared with the wild type CA (Figures 5C and 5D).

On the contrary, the GFP-expressing virus with G116E was more sensitive to humanTRIM5α expressed from the SeV in CEMss cells than the wild type virus (Figure 5B, right). These results again confirmed the results in the live virus replication experiments shown in Figure 4. In the case of TK-ts13, cells stably expressing human TRIM5α in which TRIM5α expression is in more physiological levels; however, the difference in sensitivity to human TRIM5α between the wild type and G116E lentivector was not observed (Figure 5C and 5D). Furthermore, when we used TRIM5α knockout Jurkat cells, we also failed to detect the difference in sensitivity to human TRIM5α between the wild type and G116E virus (data not shown). These results indicated that the effect of G116E substitution is virtually negligible at physiological levels of endogenous human TRIM5α, although this substitution increases the susceptibility of HIV-1 to human TRIM5α.

### A G-to-E substitution at the 116th position did not affect the association between CA and CypA or Gag processing

**Figure 6:**
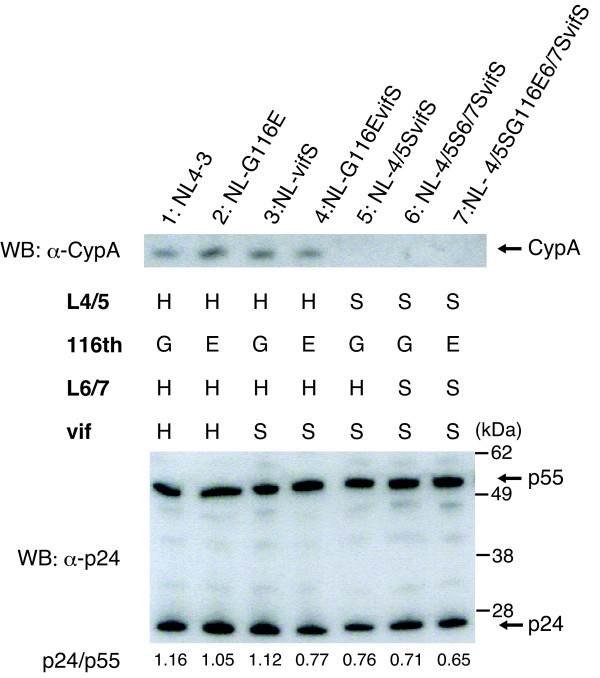
**Western blot analysis of the CA and cyclophilin A (CypA) in particles of HIV-1 derivatives**. Viral particles of the indicated HIV-1 derivatives were purified by ultracentrifugation through a 20% sucrose cushion. CypA, p24, and p55 proteins were visualized by Western blotting (WB) using anti-CypA and anti-p24 antibody, respectively. "H" and "S" denote the amino acid sequences derived from HIV-1 and SIVmac239, respectively. The ratio of the amount of p24 to that of p55 of each virus is shown at the bottom. A representative of three independent experiments is shown.

To clarify whether the 116th amino acid substitution affects the association of CypA with CA, the CypA content in the wild type and mutant virions was evaluated by Western blot analysis. As shown in Figure 6, CypA was detected in virions with HIV-1 L4/5 (lanes 1 to 4, upper panel), but not in those with SIVmac L4/5 (lanes 5 to 7) indicating that the G-to-E substitution at the 116th amino acid position had no effect on CypA binding of HIV-1 CA. When we used anti-p24 antibody (Figure 6, lower panel), p55 Gag precursors and mature p24 CA were detected. The HIV-1 Gag precursor proteins with SIVmac L4/5 and L6/7 were processed nearly normally in the virion, although there were slight differences in the ratios of p24 to p55 among HIV-1 derivatives (Figure 6, bottom). In particular, the virus with SIVmac L4/5 and L6/7 tended to contain increased amount of p55 Gag precursors (lane 6, bottom); however, addition of G116E substitution did not facilitate the cleavage of Gag (lane 7).

### Structural model of the capsid protein

**Figure 7:**
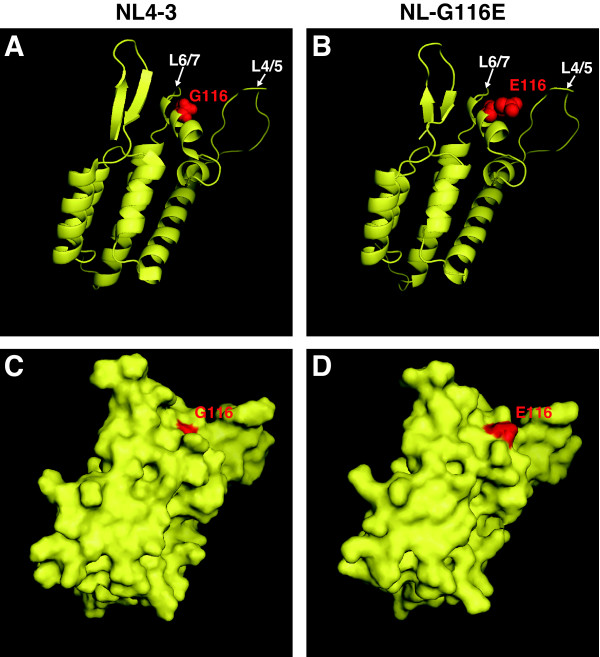
**Structural model of the N-terminal domain of HIV-1 CA with G116E substitution**. Panel A shows the template structure of the N-terminal domain of the HIV-1 CA; panel B shows the model of the domain structure with the G116E mutation. The ribbons represent the protein backbone; G and E on the 116^th ^position with their side chains are shown in red spheres. Panels C and D show surface views of the template and model structures respectively with the 116^th ^position indicated in red. The loops between α-helices 4 and 5 (L4/5) and 6 and 7 (L6/7) are labeled.

To obtain further insight into the effects of the G-to-E single amino acid substitution at the 116th position of the CA on its three-dimensional (3-D) structure, the 3-D model of the N-terminus of the CA was constructed by homology-modeling on the basis of the published crystal structure of the N-terminus of the CA of NL4-3 (PDB number 1GWP) [29] (Figure 7). Position 116 is located in the 6^th ^helix near the L4/5 and L6/7 and is apparently exposed to the surface of the protein (Figure 7 upper panels). The substitution of G to E might be important because in contrast to G, which lacks a side chain, E has a long side chain with a negative charge (Figure 7 lower panels). The mutation can therefore have two possible effects. First, if the residue is located in the interaction site, it can change the local complementarity between CA and TRIM5α. Second, even if the residue is not directly in the binding site, the change in the side chain and polarity can influence the configuration of nearby loops and, thereby, influence a binding site that is located somewhere else on the protein. Notably, the loops being flexible parts of the protein are slightly repositioned in the modeled structure with G116E substitution (Figure 7A and 7B).

## Discussion

By long-term cultivation of human CEMss cells infected with NL-ScaVR6/7S (NL-4/5S6/7SvifS), a simian tropic HIV-1 that could grow efficiently in CM cells but inefficiently in human cells, we succeeded in rescuing the impaired replicative capability of the virus in human cells. Sequence analysis of the MA-CA region of the adapted virus revealed that the there was a G-to-E single amino acid substitution at the 116th position of the CA. Introduction of this substitution into the molecular DNA clone of NL-4/5S6/7SvifS indeed improved the virus' replicative capability in human cells. We thus concluded that the recovered replicative capability in human cells was mainly the result of acquisition of the single amino acid substitution at the 116th position of the CA, although small effects of mutations in regions other than the MA-CA cannot be fully excluded at present.

Although the 116th position of the CA is highly variable among natural HIV-1 strains from subtypes A to K, no virus with E at the 116th position was found in the Los Alamos HIV sequence database 2009 http://www.hiv.lanl.gov/. On the other hand, most HIV-2 and SIVmac strains have glutamine, which has a long side chain similar to E, at this position, and some strains have E. It is possible that the combination of the amino acid residue at the 116th position and L6/7 is important for viral growth. Consistent with this hypothesis, NL-4/5SG116EvifS, a virus with an HIV-1 derived L6/7 and the G116E substitution, showed impaired growth in MT4 cells (data not shown).

The precise reasons for the impaired replicative capability of NL-4/5S6/7SvifS and effect of G116E in human cells remain to be elucidated. Analysis of a series of CA mutants shown in Figures 4 and 5 clearly excluded the possibility that the impaired replicative capability of NL-4/5S6/7SvifS in human cells resulted from an increased sensitivity to human TRIM5α because a virus with the SIVmac L4/5 and L6/7 (4/5S6/7S) showed similar infectivity to the wild-type virus in the presence of human TRIM5α, and a virus with the SIVmac L4/5, L6/7, and G116E substitution (4/5SG116E6/7S) became more sensitive to human TRIM5α (Figure 5B). On the other hand, the virus with the SIVmac L4/5 and L6/7 showed slightly impaired cleavage of p55 Gag precursors, although p24 mature CA proteins were clearly detected (Figure 6). However, the addition of G116E substitution did not facilitate the cleavage of Gag, and a small defect in Gag processing could only partially explain the attenuated growth of NL-4/5S6/7SvifS. Another possibility is that NL-4/5S6/7SvifS was restricted by a certain intrinsic restriction factor that was previously suggested to be present in human cells [13,14], and that the adapted virus could escape from this restriction by G116E substitution, since the G116E was acquired through the adaptation in human cells. It is thus necessary to conduct further analysis to substantiate this unidentified restriction factor.

Although the G116E substitution occurred during long-term cultivation of human cells infected with NL-4/5S6/7SvifS, the viruses with G116E unexpectedly became resistant to CM TRIM5α-mediated restriction (Figures 4 and 5). Replacing the HIV-1 L6/7 (HNPPIP) of the CA with that of SIVmac239 (RQQNPIP) resulted in elongation of the loop by one amino acid, and it is reasonable to assume that the G116E substitution occurred to compensate the structural warp caused by the extended L6/7. This compensatory substitution occurred at the central position of the surface composed of L4/5 and L6/7, a structure considered to be important for TRIM5α binding [24]. The amino acid substitution of G with E at the 116^th ^position caused an important change in the structure of the surface composed of L4/5 and L6/7 because G, which has no side chain, was replaced by E, which has a long, negatively charged side chain as shown in Figure 7. This change in the conformational structure of L4/5 and L6/7 might affect the interaction between the CA and TRIM5α. Alternatively, this single amino acid substitution might influence the configuration of surrounding loops by the changes in the side chain and polarity without directly involving the binding site of TRIM5α.

## Conclusion

We succeeded in rescuing the impaired replicative capability of simian tropic HIV-1 NL-4/5S6/7SvifS and unexpectedly identified a single amino acid substitution in the CA that affects viral sensitivity to CM TRIM5α-mediated restriction. This finding will increase our understanding of the detailed molecular interactions between the CA and TRIM5α.

## Abbreviations

HIV-1: human immunodeficiency virus type 1; SIVmac: simian immunodeficiency virus isolated form macaque; CM: cynomolgus monkey; Rh: rhesus monkey; SHIV: HIV-1/SIV chimeric virus; CypA: cyclophilin A; TRIM: tripartite motif; CA: capsid; GFP: green fluorescence protein; VSV-G: vesicular stomatitis virus glycoprotein; SeV: Sendai virus; L4/5: a loop between α-helices 4 and 5; L6/7: a loop between α-helices 6 and 7.

## Competing interests

The authors declare that they have no competing interests.

## Authors' contributions

AK, and EEN performed the in vitro experiments; KB performed computational modeling of CA protein; and AK, TS, KB and EEN wrote the paper.
